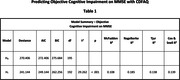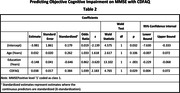# Predicting Objective Cognitive Impairment on MMSE with CDFAQ

**DOI:** 10.1002/alz.091950

**Published:** 2025-01-03

**Authors:** Caio Peixoto Tavares, Isabelle de Aguiar Maia, Giovanna Correia Pereira Moro, Aline Siqueira de Souza, Jéssica Diniz Ferreira, Ivonne Carolina Bolaños Burgos, Gabriela Tomé Oliveira Engelmann, Maissa Ferreira Diniz, Marco Aurélio Romano‐Silva, Jonas Jardim de Paula, Maria Aparecida Camargos Bicalho, Bernardo de Mattos Viana

**Affiliations:** ^1^ Universidade Federal de Minas Gerais, Belo Horizonte, Minas Gerais Brazil; ^2^ Older Adult’s Psychiatry and Psychology Extension Program I Federal University of Minas Gerais, Belo Horizonte, Minas Gerais Brazil; ^3^ Older Adult’s Psychiatry and Psychology Extension Program (PROEPSI), School of Medicine, Universidade Federal de Minas Gerais (UFMG), Belo Horizonte, Minas Gerais Brazil; ^4^ Sciences Applied to Adult Health Postgraduate Program, School of Medicine, Universidade Federal de Minas Gerais (UFMG), Belo Horizonte, Minas Gerais Brazil; ^5^ Cog‐Aging Research Group, Belo Horizonte, Minas Gerais Brazil; ^6^ INCT – NeuroTecR and CTMM, Belo Horizonte, Minas Gerais Brazil; ^7^ Molecular Medicine Postgraduate Program, School of Medicine, Universidade Federal de Minas Gerais (UFMG), Belo Horizonte, Minas Gerais Brazil; ^8^ Cog‐Aging Research Group, Universidade Federal de Minas Gerais (UFMG), Belo Horizonte, Minas Gerais Brazil; ^9^ Jenny de Andrade Faria Institute – Outpatient Reference Center for the Elderly, Universidade Federal de Minas Gerais (UFMG), Belo Horizonte, Minas Gerais Brazil; ^10^ Molecular Medicine Postgraduate Program, Faculty of Medicine, Universidade Federal de Minas Gerais (UFMG, Belo Horizonte, Minas Gerais Brazil; ^11^ Department of Psychiatry, School of Medicine, Federal University of Minas Gerais, Belo Horizonte, Minas Gerais Brazil; ^12^ Molecular Medicine Program, Faculdade de Medicina, Belo Horizonte, Minas Gerais Brazil; ^13^ National Institute of Science and Technology Neurotec R (INCT‐MM), Belo Horizonte, Minas Gerais Brazil; ^14^ Department of Clinical Medicine, Faculdade de Medicina, Universidade Federal de Minas Gerais, Belo Horizonte, Minas Gerais Brazil

## Abstract

**Background:**

The Cognitive Domains and Functional Assessment Questionnaire (CDFAQ) assess cognitive and functional decline for Neurocognitive Disorders based on the DSM‐5 criteria (1). It’s accuracy to the Informant Questionnaire on Cognitive Decline in the Elderly ‐ Long Version (IQCODE‐LV) has been assessed (2), and was translated and validated into English (3). The informant version (CDFAQ‐IV) assess: Complex Attention (CA), Executive Functions (EF), Learning and Memory (LM), Language (L), Perceptual‐Motor (PM) and Social Cognition.

**Methods:**

To predict objective cognitive impairment on Mini‐Mental State Examination (MMSE) with CDFAQ‐IV. Both instruments were applied in 196 older adults and their informants. The cut‐off points on MMSE were 19/20 for illiterates and 23/24 for those with one year or more of formal education (4). A multiple logistic regression was made to assess the CDFAQ likelihood to predict a performance below the cut‐off points on MMSE controlled by years of age and of education. This study was approved by the ethics committee of UFMG.

**Results:**

The median age of the older adults was 73 years (IQR = 13.25), median years of education of 4 years (IQR = 5), median CDFAQ‐IV score of 64 (IQR = 9), and median MMSE score of 23 (IQR = 9). One hundred and six participants (54.08%) had impaired results on MMSE. When controlled by age and education, the Odds Ratio (OR) of one point increase in CDFAQ had an OR of 1.039 (p = 0.029) to predict an objective cognitive impairment on MMSE. A one‐year increase in formal education, had a decreased OR of 0.862 (p < 0.001). Age was not associated to the prediction of an objective cognitive impairment on MMSE, when controlled by education and CDFAQ performance (p = 0.106).

**Conclusions:**

Years of education and CDFAQ scores were related to the prediction of objective cognitive impairment measured by MMSE. These are still preliminary studies, but these results point to the need of considering the importance of years of education for future norms of the CDFAQ.